# Donepezil, Anti-Alzheimer's Disease Drug, Prevents Cardiac Rupture during Acute Phase of Myocardial Infarction in Mice

**DOI:** 10.1371/journal.pone.0020629

**Published:** 2011-07-05

**Authors:** Mikihiko Arikawa, Yoshihiko Kakinuma, Takemi Handa, Fumiyasu Yamasaki, Takayuki Sato

**Affiliations:** 1 Department of Cardiovascular Control, Kochi Medical School, Nankoku, Kochi, Japan; 2 Department of Clinical Laboratory, Kochi Medical School, Nankoku, Kochi, Japan; Charité, Campus Benjamin Franklin, Germany

## Abstract

**Background:**

We have previously demonstrated that the chronic intervention in the cholinergic system by donepezil, an acetylcholinesterase inhibitor, plays a beneficial role in suppressing long-term cardiac remodeling after myocardial infarction (MI). In comparison with such a chronic effect, however, the acute effect of donepezil during an acute phase of MI remains unclear. Noticing recent findings of a cholinergic mechanism for anti-inflammatory actions, we tested the hypothesis that donepezil attenuates an acute inflammatory tissue injury following MI.

**Methods and Results:**

In isolated and activated macrophages, donepezil significantly reduced intra- and extracellular matrix metalloproteinase-9 (MMP-9). In mice with MI, despite the comparable values of heart rate and blood pressure, the donepezil-treated group showed a significantly lower incidence of cardiac rupture than the untreated group during the acute phase of MI. Immunohistochemistry revealed that MMP-9 was localized at the infarct area where a large number of inflammatory cells including macrophages infiltrated, and the expression and the enzymatic activity of MMP-9 at the left ventricular infarct area was significantly reduced in the donepezil-treated group.

**Conclusion:**

The present study suggests that donepezil inhibits the MMP-9-related acute inflammatory tissue injury in the infarcted myocardium, thereby reduces the risk of left ventricular free wall rupture during the acute phase of MI.

## Introduction

In 2000, K. J. Tracey and coworkers reported the role of efferent vagus nerve signaling in modulating inflammation [1]. The electrical stimulation of the efferent vagus nerve releases acetylcholine (ACh), a principal vagal neurotransmitter, and ACh seems to attenuate systemic inflammatory responses by inhibiting the production of pro-inflammatory cytokines from activated macrophages and other immune cells. The ‘cholinergic’ anti-inflammatory pathway would provide a new therapeutic modality for the clinical treatment of inflammatory disorders [2].

We have reported another aspect of the beneficial effect of the vagal nerve stimulation [3]. Chronic vagal nerve stimulation with an implantable stimulator prevented the cardiac pumping dysfunction and improved long-term survival in rats with chronic heart failure after large myocardial infarction (MI). Although the precise mechanisms of the protective effect of the vagal nerve stimulation on cardiomyocytes have not yet been fully elucidated, our previous studies have clearly demonstrated that the antiarrhythmogenic effects are mediated by preservation of the gap-junctional protein, connexin 43, and the antiapoptotic effects are derived from the PI3K/Akt/HIF-1α signaling pathway in cardiomyocytes, both of which are proposed to be involved in the mechanism of the cardioprotective effect of the vagal nerve stimulation [4], [5]. The therapeutic modality of the vagal nerve stimulation has been recently applied for patients with heart failure in a clinical trial without significant side effects [6]–[8], however, it might have disadvantages because it is an invasive procedure suffering a surgical strain to the patients. Since a novel and a more effective therapeutic strategy against heart failure is mandatory, we have studied the effect of an acetylcholinesterase inhibitor, donepezil, on heart failure expecting the cardioprotective effect. Donepezil is a therapeutic acetylcholinesterase inhibitor for treatment of Alzheimer's disease, a neurodegenerative disorder characterized with a depletion of nicotinic ACh receptors and a loss of cholinergic neurons [9]. A number of studies using animal models have demonstrated that donepezil possesses the neuroprotective activity *in vivo* [10], and *in vitro* [11], [12]. In addition, it has been recently demonstrated that the administration of donepezil reproduced the effect of the vagal nerve stimulation. For example, donepezil has been shown to 1) prevent cardiac remodeling with enhancing vagal activity in a rat chronic MI model [13], 2) improve survival by preventing pumping failure in a mouse volume overload model [14] and 3) up-regulate angiogenesis by modulating angiogenesis-responsible machinery of endothelial cells in a mouse ischemic hindlimb model [15], indicating that the donepezil possesses not only neuroprotective but cardioprotective effects. In comparison with such chronic effects, however, the acute effect of donepezil during an acute phase of MI remains unclear.

Even though surgical techniques for ischemic heart failure have been improved, left ventricular free wall rupture has been still one of the most lethal complications occurring in up to 10% patients with acute MI [16]. Clinical and pathological studies in humans have reported that the mean interval between diagnosis of acute MI and the appearance of left ventricular free wall rupture ranged from 1 to 5 days after MI [17]–[21]. Approximately 60% of the left ventricular free wall rupture occurred within 5 days postinfarction [22]. A large number of studies have shown that a severe inflammatory cell infiltration following acute MI markedly activates various matrix metalloproteinases (MMPs) which rapidly degrade the fibrillar collagen network and weaken the tensile strength of the infarcted myocardium, resulted in wall rupture [23], [24]. Therefore, the suppression of MMPs during an acute phase of MI is a potential target for the therapeutic intervention [25]–[30].

In the present study, noticing recent findings of a cholinergic mechanism for anti-inflammatory actions, the effect of donepezil on inflammatory response and cardiac rupture during the acute phase of MI was investigated. Present results showed that donepezil inhibits MMP-9, a member of the MMP family, in macrophages and attenuates infiltration of inflammatory cells into the infarcted myocardium, thereby reduces the risk of cardiac rupture during the acute phase of MI, suggesting that donepezil can be a new potential candidate for a clinically useful drug for heart failure therapy.

## Materials and Methods

### Animals

Male C57BL/6 mice (Japan SLC inc., Hamamatsu, Japan) aged between 9 and 11 weeks and weighed 20–25 g were used. All animal procedures were performed in strict accordance with guidelines of the Physiological Society of Japan and were approved by the Animal Research Committee of Kochi Medical School (Permit Number: C-00036).

### Macrophage isolation and sample preparation

From mice euthanized with ether inhalation, peritoneal macrophages were obtained by washing out the abdominal cavity with ice-cold phosphate buffered saline (PBS). Cells were collected by centrifugation, resuspended in RPMI 1640 medium (Sigma-Aldrich Japan, Tokyo, Japan) containing 10% fetal bovine serum (Tissue Culture Biologicals, CA, USA), and seeded at a concentration of more than 3×10^6^ cells/well of a 12-well plastic dish. After incubation at 37°C for 2 hrs, non-adherent cells were flushed away and adhered cells were further incubated at 37°C for 18 hrs in RPMI 1640 medium with or without donepezil, an acetylcholinesterase inhibitor, provided by Eisai Co., Ltd. (Tokyo, Japan) at a concentration of 100 µM. Three hours after the addition of lipopolysaccaride (LPS, Sigma-Aldrich Japan, Tokyo, Japan) at a concentration of 10 ng/ml, culture medium was collected from each well and mixed with same amount of twice concentrated sample buffer (125 mM Tris-HCl (pH 6.8), 20% glycerol, 4% sodium dodecyl sulfate (SDS), 10% 2-mercaptoethanol and 0.1% bromophenol blue), or the sample buffer was directly added to the adhered cells. Protein samples were boiled with a THERMO BLOCK ND-M11 (Nissin, Tokyo, Japan) for 5 min before electrophoresis.

### Immunocytochemistry

Isolated macrophages seeded onto a glass-bottom dish (Matsunami Glass Industry, Ltd., Tokyo, Japan) were stimulated by LPS with or without donepezil pretreatment as described above. Cells fixed with 4% paraformaldehyde phosphate buffer solution for 30 min at room temperature were permeabilized with 1% buffered triton X-100 for 15 min, and blocked with 10% goat serum in tris buffered saline with 0.2% Tween 20 (TBS-T) for 30 min. Thereafter, cells were incubated with a rabbit polyclonal anti mouse matrix metalloproteinase-9 (MMP-9) antibody (Abcam, Tokyo, Japan) diluted 1∶500 for 60 min and a fluorescent conjugated goat anti rabbit IgG antibody (Invitrogen, Tokyo, Japan) diluted 1∶1000 for 45 min at room temperature. After nuclei were counterstained with Hoechst 33258 (Invitrogen, Tokyo, Japan), fluorescence was visualized and photographed with a confocal laser scanning microscope FV-300 with a fluoview software version 4.3 (Olympus, Tokyo, Japan).

### Animal model and drug administration

After induction of anesthesia by intraperitoneal injection of pentobarbital (50 mg/kg), mice were put on a heated pad and artificially ventilated (5% carbon dioxide and 95% oxygen) with a volume-controlled MiniVent Model 845 Ventilator for mice (Harvard Apparatus, MA, USA) with a stroke volume of 300 µL and a respiratory rate of 130 breaths/min. Under a surgical microscope (Leica M6541, Leica Microsystems, Tokyo, Japan), a left-side thoracotomy was performed and a pericardium was partially stripped to expose the heart. The left anterior descending coronary artery was ligated with an 8-0 silk suture (Akiyama MEDICAL MFG, Tokyo, Japan). Myocardial ischemia was confirmed by blanching of the anterior wall of the left ventricle and ST segment elevation on the electrocardiogram. In sham-operated mice, the same surgical procedures were performed except for the ligation of the coronary artery. After the chest was closed with a 5-0 polyester suture (Bear Medic, Tokyo, Japan), animals were kept warm under a heat lamp during the recovery period. Infarcted mice were randomly divided into untreated and donepezil-treated groups. Donepezil was administrated orally at dosage of 5.0 mg/kg/day. Mice recovered from the surgical damage were housed under identical conditions within 6 hrs after surgery and given food and water or drug *ad libitum*. The cages were inspected daily for morbidity and mortality, and autopsy was immediately performed to determine the cause of death in all dead animals. Cardiac rupture was confirmed based on a diagnosis of the presence of a large amount of blood clot within the chest cavity or the wall perforation at the left ventricular infarct area, and a rupture rate was calculated in percentage as a number of ruptured animals divided by a number of total operated animals in each group.

### Heart rate and blood pressure measurement

At 3 days after surgery, sham-operated and infarcted mice were kept in a preheated dark chamber for at least 10 min in order to increase blood flow to the tail. The mouse tail was inserted through an appropriate cuff, and heart rate (HR) and systolic blood pressure (SBP) were measured noninvasively by a computer-automated tail-cuff system with a data analysis software (BP-98A, Softron, Tokyo, Japan) according to the manufacturer's instructions. HR and SBP were finally determined by averaging the data obtained from 5 to 10 successful measurement cycles.

### Histology and immunohistochemistry

Mice were anesthetized and sacrificed at appropriate time points after surgery. Hearts were immediately excised and washed with ice-cold PBS. Paraformaldehyde-fixed and paraffin-embedded heart samples were sectioned transversely at 3–5 µm thick at a middle part of the left ventricular infarct area. After dewaxed and rehydrated by sequential treatment with xylene and a graded ethanol series, sections were routinely stained with hematoxylin and eosin (HE) or Masson's trichrome staining. Histological images were captured with a digital camera and wall thickness was measured. Infarct size was calculated in percentage as total infarct circumference divided by total LV circumference according to the method of Pfeffer [31], [32]. Under high power field magnification (×400), the number of nuclei at the border zone of the infarct was manually counted from randomly selected areas (4 areas/section), and expressed as nuclei per square millimeter. For immunohistochemistry, deparaffinized and rehydrated sections were heated at 95°C for 30 min in a Tris-EDTA buffer (Target Retrieval Solution, pH 9, DakoCytomation, Denmark) for antigen retrieval. After a 45 min blocking step with 10% goat serum in TBS-T, sections were incubated with a rabbit polyclonal anti mouse MMP-9 antibody (Abcam, Tokyo, Japan) diluted 1∶100 for 90 min and a fluorescent conjugated goat anti rabbit IgG antibody (Invitrogen, Tokyo, Japan) diluted 1∶500 for 45 min at room temperature. Sections were counterstained for nuclei with Hoechst 33258 (Invitrogen, Tokyo, Japan). Observations were made with a confocal laser scanning microscope FV-300 (Olympus, Tokyo, Japan).

### RT-PCR

From excised hearts, right ventricle and left ventricle including septum were dissected. The left ventricle of the infarcted heart was further separated into infarct and non-infarct area. Infarct tissues were frozen with liquid nitrogen and stored at −80°C until use. Total RNA was isolated from tissues by phenol/chloroform extraction method using TRIzol Reagent (Invitrogen Japan, Tokyo, Japan) according to the manufacturer's instructions. The Reverse Transcriptase XL for RT-PCR kit (Takara, Japan) was used to obtain first-strand cDNA, which was then amplified with gene specific primers by a PCR thermal cycler (TP-600, Takara, Japan). For mouse MMP-9, a sense primer (5′-GCATACTTGTACCGCTATGG-3′) and an antisense primer (5′-TAACCGGAGGTGCAAACTGG-3′) were used. Products were electrophoretically fractionated on 2% agarose gels and photographed by Kodak Gel Logic 100 Imaging System and Kodak Molecular Imaging Software v. 4. 5. 0.

### Electrophoresis and immunoblotting

The left ventricular infarct area was carefully dissected from an excised heart under a surgical microscope and homogenized in an extraction buffer containing 50 mM Tris-HCl (pH 6.8), 150 mM NaCl, 1% Triton X-100 and 10% SDS. After centrifugation, the supernatants were mixed with same amount of twice concentrated sample buffer and boiled for 5 min. Protein samples obtained from *in vivo* and *in vitro* studies were separated by SDS-polyacrylamide gel electrophoresis (SDS-PAGE) according to the method of Laemmli [33]. For immunoblotting analysis, proteins were electrophoretically transferred to a PVDF-membrane (Immobilon-P, Millipore Japan, Tokyo, Japan) and blocked in a blocking buffer containing 5% dehydrated skim milk (Difco Laboratories Inc., MI, USA) for at least 30 min at room temperature or overnight at 4°C. The membrane was incubated with a rabbit anti mouse MMP-9 antibody (Abcam, Tokyo, Japan) diluted 1∶2000 for 90 min, followed by incubation with a horseradish peroxidase (HRP)-conjugated goat anti rabbit IgG antibody (Invitrogen, Tokyo, Japan) diluted 1∶5000 for 45 min at room temperature. HRP activity was detected by chemiluminescence using an ECL plus Western Blotting Detection System (GE Healthcare Japan Corporation, Tokyo, Japan).

### Gelatin zymography

The enzymatic activity of MMP-9 was measured by gelatin zymography under nonreducing conditions according to the standard procedures. Briefly, macrophage culture media or tissue lysate of left ventricular infarct area were mixed with same amount of twice concentrated sample buffer which does not contain 2-mercaptoethanol, and stored at 4°C for overnight without boiling. The samples were then electrophoretically separated on a polyacrylamide gel containing 1 mg/ml gelatin as a substrate. The gel was washed with 2.5% Triton X-100 solution for 60 min at room temperature, and then incubated with a developing buffer containing 50 mM Tris-HCl (pH 7.5), 200 mM NaCl, 10 mM CaCl_2_, 1 µM ZnCl_2_ and 0.02% NaN_3_ for 20 hrs at 37°C. The gel was stained with 0.5% Coomassie Brilliant Blue R-250 for 30 min and destained for appropriate time until clear bands were visualized, and was scanned by a digital scanner (ES-2000, Epson Co., Tokyo, Japan).

### Statistical analysis

Data are presented either as means ± SD or a percentage compared with a control. Nonparametric comparisons between two groups were performed with a Mann-Whitney U-test. Multiple comparisons among groups were performed with a nonparametric one-way ANOVA using a Kruskal-Wallis test. The mortality data were analyzed by a Chi-square test. A value of *P<0.05* was considered statistically significant.

## Results

### Effect of donepezil on macrophage MMP-9

**Figure 1:**
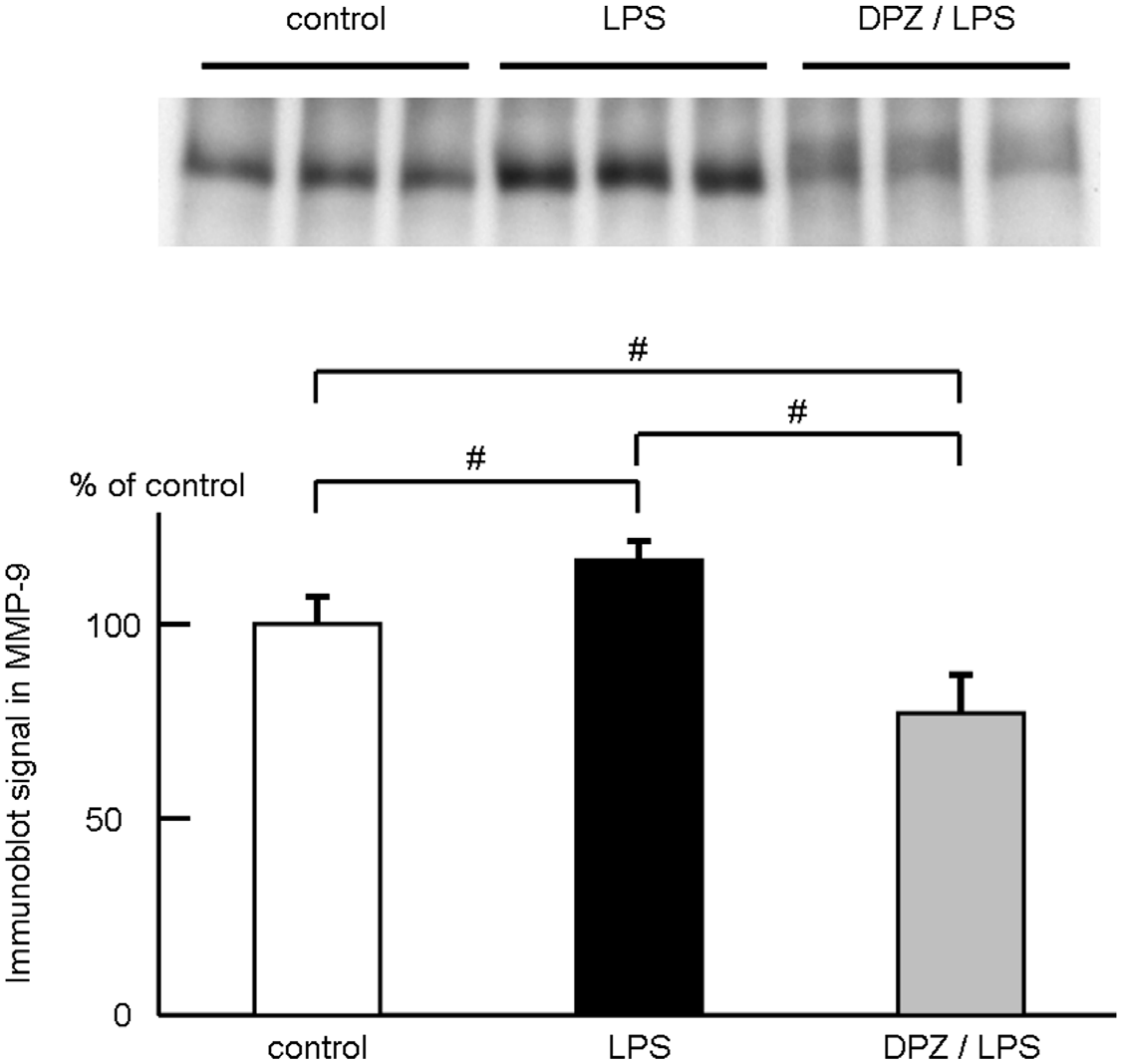
Effect of donepezil on MMP-9 in macrophages. MMP-9 in culture medium was evaluated by Western blot analysis before and after LPS (10 ng/ml) treatment with or without donepezil pretreatment (100 µM). Compared to control (control), LPS treatment significantly increased MMP-9 in culture medium (LPS), whereas donepezil pretreatment significantly decreased MMP-9 even in the presence of LPS (DPZ/LPS). #, *P<0.01*.

**Figure 2:**
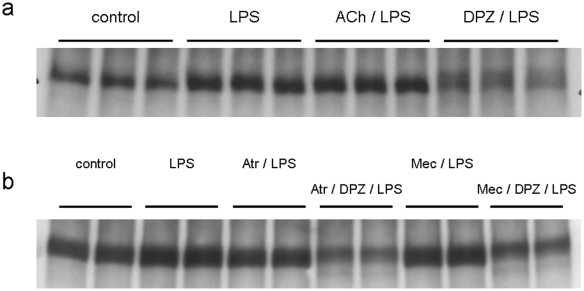
The involvement of ACh in the inhibitory effect of donepezil on macrophage MMP-9. (a) LPS treatment increased MMP-9 (LPS) in culture medium, which was inhibited by donepezil pretreatment (DPZ/LPS). However, ACh could not reproduce the inhibitory effect of donepezil (ACh/LPS). (b) Donepezil inhibited the LPS-induced MMP-9 even in the presence of a muscarinic ACh receptor blocker, atropine (Atr/DPZ/LPS), or a nicotinic ACh receptor blocker, mecamylamine (Mec/DPZ/LPS). Both blockers showed no effect on LPS-induced MMP-9 independently (Atr/LPS and Mec/LPS).

**Figure 3:**
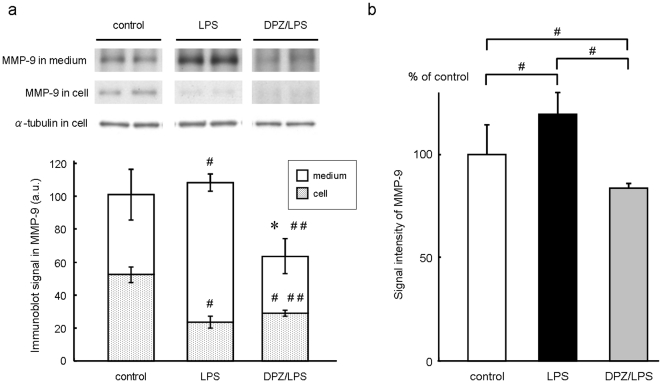
Western blot and zymographic analysis on macrophage MMP-9. (a) Western blot analysis of MMP-9 content in culture media and macrophage cells before and after LPS treatment with or without donepezil pretreatment. In control (control), MMP-9 was stored within cells and also secreted to culture medium. In LPS-treated cells (LPS), MMP-9 was significantly decreased within cells and increased in culture medium. In donepezil-pretreated and LPS-treated cells (DPZ/LPS), MMP-9 within both cells and medium were as low as undetectable level. The expression of α-tubulin was shown as an internal control. #, *P<0.01* versus control. ##, *P<0.01* versus LPS. *, *P<0.05* versus control. (b) Gelatin zymography for analysis of MMP-9 enzymatic activity. Compared to control (control), proteinase activity of MMP-9 secreted to culture medium was significantly high in LPS-treated cells (LPS) and low in donepezil-pretreated and LPS-treated cells (DPZ/LPS). #, *P<0.01*.

Compared to the control (control, 100.00±7.08%, n = 11), lipopolysaccaride (LPS) treatment at a concentration of 10 ng/ml for 3 hrs significantly increased macrophage matrix metalloproteinase-9 (MMP-9) secretion to culture medium (LPS, 116.16±5.37% versus control, n = 11, *P<0.01*). However, MMP-9 in culture medium of the donepezil-pretreated macrophages was significantly lower than the control (DPZ/LPS, 76.90±10.92% versus control, n = 11, *P<0.01*). Consequently, donepezil pretreatment reduced the LPS-induced MMP-9 secretion from macrophages by 33.80% (*P<0.01*, Figure 1). In this study, we used donepezil expecting its inhibitory action on acetylcholinesterase. Therefore, the effect of ACh on macrophage MMP-9 was also investigated. However, unexpectedly, the LPS-induced increase of MMP-9 was not inhibited by pretreatment of ACh (100 µM) (ACh/LPS in Figure 2a). Moreover, the MMP-9 inhibition by donepezil was observed even in the presence of a muscarinic ACh receptor blocker, atropine (100 µM) or a nicotinic ACh receptor blocker, mecamylamine (5 µM) (Atr/DPZ/LPS and Mec/DPZ/LPS in Figure 2b). These results indicate that the inhibitory effect of donepezil on macrophage MMP-9 is independent of ACh. The alteration of MMP-9 content within cells before and after LPS treatment with or without donepezil pretreatment was examined (Figure 3a). In the control (control), MMP-9 was stored within cells and also secreted to culture medium (cell, 52.5±4.7 in a.u., n = 6 and medium, 48.5±15.5 in a.u., n = 6). When cells were treated with LPS for 3 hrs (LPS), MMP-9 was significantly decreased in cells (23.6±3.8 in a.u., n = 6, *P<0.01* versus control) and increased in culture medium (84.9±5.2 in a.u., n = 6, *P<0.01* versus control), indicating that LPS treatment induced secretion of MMP-9 from cells to culture medium. In the case of cells pretreated with donepezil (DPZ/LPS), intracellular MMP-9 was undetectable levels like that in donepezil-pretreated culture medium (cell, 29.1±1.9 in a.u., n = 6, *P<0.01* versus control, *P<0.01* versus LPS and medium, 34.4±10.5 in a.u., n = 6, *P<0.05* versus control, *P<0.01* versus LPS). It was also observed that donepezil pretreatment reduced MMP-9 content within cells not treated with LPS (data not shown). These results indicate that donepezil suppresses the de novo synthesis of MMP-9 during the pretreatment period, resulted in the lower content of MMP-9 in the culture medium even after the LPS treatment. Gelatin zymography was carried out to analyze the enzymatic activity of MMP-9 secreted to culture medium (Figure 3b). Although pro- and active-forms of MMP-9 could not be clearly distinguished, MMP-9 retained its proteinase activity even after being secreted to culture medium. The signal intensity of MMP-9 was significantly high in LPS-treated cells (LPS, 119.33±10.73% versus control, n = 6, *P<0.01*) and low in donepezil-pretreated and LPS-treated cells (DPZ/LPS, 83.52±2.59% versus control, n = 6, *P<0.01*), compared to control (control, 100.00±14.3%, n = 6).

**Figure 4:**
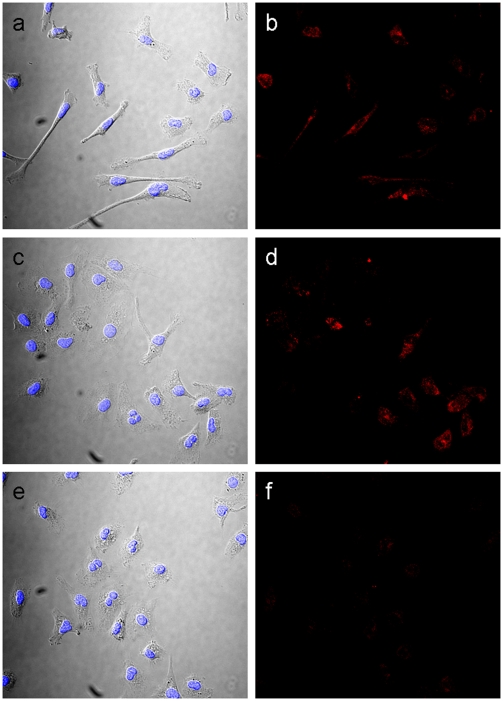
Immunocytochemistry with anti MMP-9 antibody in macrophages. DIC images showed that control macrophages had a spindle shape (a), whereas LPS-treated macrophages had a relatively round shape and numerous intracellular vacuoles regardless of donepezil pretreatment (c and e). Immunofluorescence micrographs showed that MMP-9 signals were observed around cytoplasm in the control (b) and the LPS-treated cells (d), which was lowered to undetectable levels by donepezil pretreatment (f).

The effect of donepezil on the macrophage MMP-9 was further examined by immunocytochemistry (Figure 4). Isolated and cultured macrophages were observed to attach well to a substratum and form widespreading pseudopodia. Compared to the control that showed a high proportion of cells with spindle shapes (Figure 4a), LPS-treated cells displayed a relatively round shape and a numerous intracellular vacuoles (Figure 4c). Such morphological alterations induced by LPS treatment were also observed in donepezil-pretreated cells (Figure 4e), indicating that the donepezil pretreatment had no effect on the macrophage reactivity to LPS treatment. Immunofluorescence microscopy with anti MMP-9 antibody showed that MMP-9 was distributed around cytoplasm in control and LPS-treated cells (Figures 4b and d), whereas donepezil decreased the immunoreactivities of MMP-9 within cytoplasm (Figure 4f). These results indicate that the donepezil pretreatment decreased MMP-9 content within the cell, which coincided with the results obtained immunoblot analysis shown in Figure 3a.

### Effect of donepezil on risk of cardiac rupture after myocardial infarction

A total of 330 mice was operated (310 mice were subjected to coronary artery ligation, whereas 20 mice underwent sham operation) and 60 mice (18.2%) died within 24 hrs due to surgical reasons. No deaths were observed in the sham-operated mice. Ten mice (3.7%) that died during the follow up period (4 days postinfarction) without diagnosis of cardiac rupture and 37 mice (13.7%) that had obviously small left ventricular infarction were excluded from the study. Consequently, 223 mice were allocated into examining an incidence of rupture, heart rate (HR) and systolic blood pressure (SBP) measurement, histology, immunohistochemistry, and tissue collection for RT-PCR, Western blot analysis and gelatin zymography. Surviving mice with myocardial infarction (MI) were divided into 2 groups; untreated and donepezil-treated (DPZ) groups.

HR and SBP were measured noninvasively using a computer-automated tail-cuff system in sham-operated and infarcted mice at day 3 after surgery. As shown in Table 1, there was no significant difference in HR among all groups of mice (sham, 493±70 bpm, n = 6; untreated, 548±53 bpm, n = 9 and DPZ, 528±43 bpm, n = 9, N.S.). On the other hand, SBP was decreased significantly in infarcted groups (untreated, 81.6±7.4 mmHg, n = 9, *P<0.01* and DPZ, 78.3±7.9 mmHg, n = 9, *P<0.01*) compared with the sham group (98.9±5.0 mmHg, n = 6), indicating a progression of cardiac dysfunction after MI. However, values of the decreased SBP in the infarcted groups were comparable. In another set of experiments showed that no significant differences in HR and SBP were observed in normal mice treated with donepezil (DPZ, 5.0 mg/kg/day) for 3 days (HR, 504±36 bpm, n = 6 and SBP, 103.7±9.8 mmHg, n = 6). These results indicate that the donepezil administration at the dosage of 5.0 mg/kg/day did not affect HR and SBP in normal and infarcted mice. When mice were sacrificed at 3 days after MI, all excised hearts were weighed. The ratio of heart weight to body weight (HW/BW) was significantly increased in infarcted groups (untreated, 5.85±0.66 mg/g, n = 11, *P<0.01* and DPZ, 5.60±0.39 mg/g, n = 11, *P<0.01*) compared with the sham group (4.31±0.25 mg/g, n = 7). However, no significant difference was found in HW/BW among infarcted groups, suggesting that donepezil has no effect on the postinfarct cardiac enlargement during the acute phase of MI (Table 1).

**Table 1:** Physiological characteristics in sham operated and infarcted mice at 3 days after MI.

	sham	infarcted	
		untreated	DPZ
n	6	9	9
HR (bpm)	493±70	548±53	528±43
SBP (mmHg)	98.9±5.0	81.6±7.4 #	78.3±7.9 #
n	7	11	11
HW/BW (mg/g)	4.31±0.25	5.85±0.66 #	5.60±0.39 #

Values are means ± SD. HR, heart rate; SBP, systolic blood pressure; HW/BW, heart weight to body weight ratio; DPZ, donepezil-treated group. #, *P<0.01* versus sham group.

Histological analysis was performed on HE stained heart sections at 3 days after MI (Table 2). Although wall thickness of left ventricle and septum were not significantly different between untreated and donepezil-treated groups, the ratio of these in the donepezil-treated group was significantly greater than that in the untreated group (untreated, 0.51±0.07, n = 5 and DPZ, 0.69±0.20, n = 5, *P<0.05*). On the contrary, right ventricular wall thickness in the donepezil-treated group was significantly thinner than that of the untreated group (untreated, 56.1±8.91 µm, n = 5 and DPZ, 43.9±7.31 µm, n = 5, *P<0.05*). Infarct size was comparable between two groups. In the heart sections at 3 days after MI, it was observed that cardiac myocytes at mid-infarct area became necrotic and a large number of inflammatory cells infiltrated into infarct border zone. Under high power field magnification, nuclear density at the border zone of the infarct was counted and was significantly low in the donepezil-treated group compared to the untreated group (untreated, 4.42±0.36×10^3^/mm^2^, n = 5 and DPZ, 3.50±0.29×10^3^/mm^2^, n = 5, *P<0.01*).

**Table 2:** Histological analysis in heart sections at 3 days after MI.

		untreated	DPZ
n		5	5
Wall thickness	left ventricle (µm)	68.7±10.7	89.8±21.5
	septum (µm)	134.6±13.1	132.0±6.2
	left ventricle/septum	0.51±0.07	0.69±0.20 ##
	right ventricle (µm)	56.1±8.91	43.9±7.31 ##
Infarct size (%)		68.3±3.12	62.6±9.61
Nuclear density (×10^3^/mm^2^)		4.42±0.36	3.50±0.29 #

Values are means ± SD. DPZ, donepezil-treated group. #, *P<0.01*. ##, *P<0.05*.

**Figure 5:**
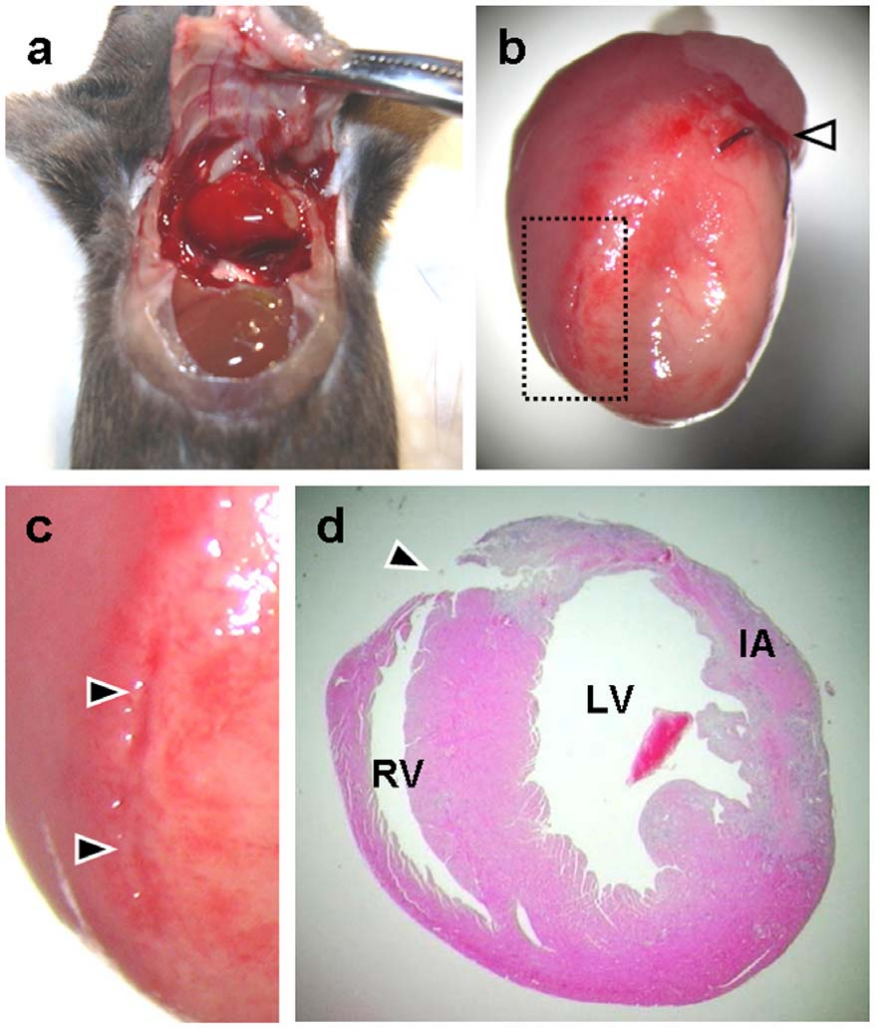
Light micrographs of an autopsied mouse that died due to cardiac rupture. (a) An infarcted mouse after the incidence of cardiac rupture. The chest cavity was filled with a large amount of clotted blood surrounding the heart. (b) The heart excised from the mouse. The ischemic area was visually identified by the pale myocardium. A white arrowhead represents the position of the coronary artery ligation. (c) An enlarged micrograph of the area shown by the small rectangle with a broken line in (b). A left ventricular perforation tends to occur at the border zone between infarcted and non-infarcted myocardium (black arrowheads). (d) The ruptured heart sectioned at a middle part of the left ventricular infarct area. The tissue was stained with hematoxylin and eosin. A black arrowhead shows the position of wall perforation occurred at left ventricular infarct border zone. RV, right ventricle; LV, left ventricle; IA, infarct area.

Within 2 days after surgery, no mice were dead in all groups. Thereafter, infarcted mice began to die due to cardiac rupture at day 3 and the numbers of those mice were further increased at day 4 after MI. As described in Methods, all dead mice were immediately autopsied to determine the cause of death, and cardiac rupture was confirmed based on a diagnosis of the presence of a large amount of blood clot within the chest cavity (Figure 5a) or a wall perforation at a left ventricular infarct area (Figures 5b and c). The wall perforation tended to occur more frequently in a central region of an infarcted left ventricle or a border zone between infarcted and non-infarcted myocardium, where wall strength became relatively weak (Figure 5d). At both 3 and 4 days after MI, a greater number of animals died with rupture in the untreated group than in the donepezil-treated group, and the rupture rate of the untreated group was reached to 30.6%, whereas the donepezil-treated group showed a lower incidence of rupture (8.7%). Statistical analysis showed that, despite the comparable values of HR and SBP at day 3 after MI among infarcted groups, donepezil administration to infarcted mice significantly decreased the incidence of rupture during the acute phase of MI (*P<0.01*, Table 3).

**Table 3:** Cardiac rupture rate at 4 days after MI.

	sham	infarcted	
		untreated	DPZ
operated	6	49	46
ruptured	0	15	4
rupture rate (%)	0	30.6	8.7 #

DPZ, donepezil-treated group. #, *P<0.01* versus untreated group.

### Effect of donepezil on MMP-9 in left ventricular infarct area

**Figure 6:**
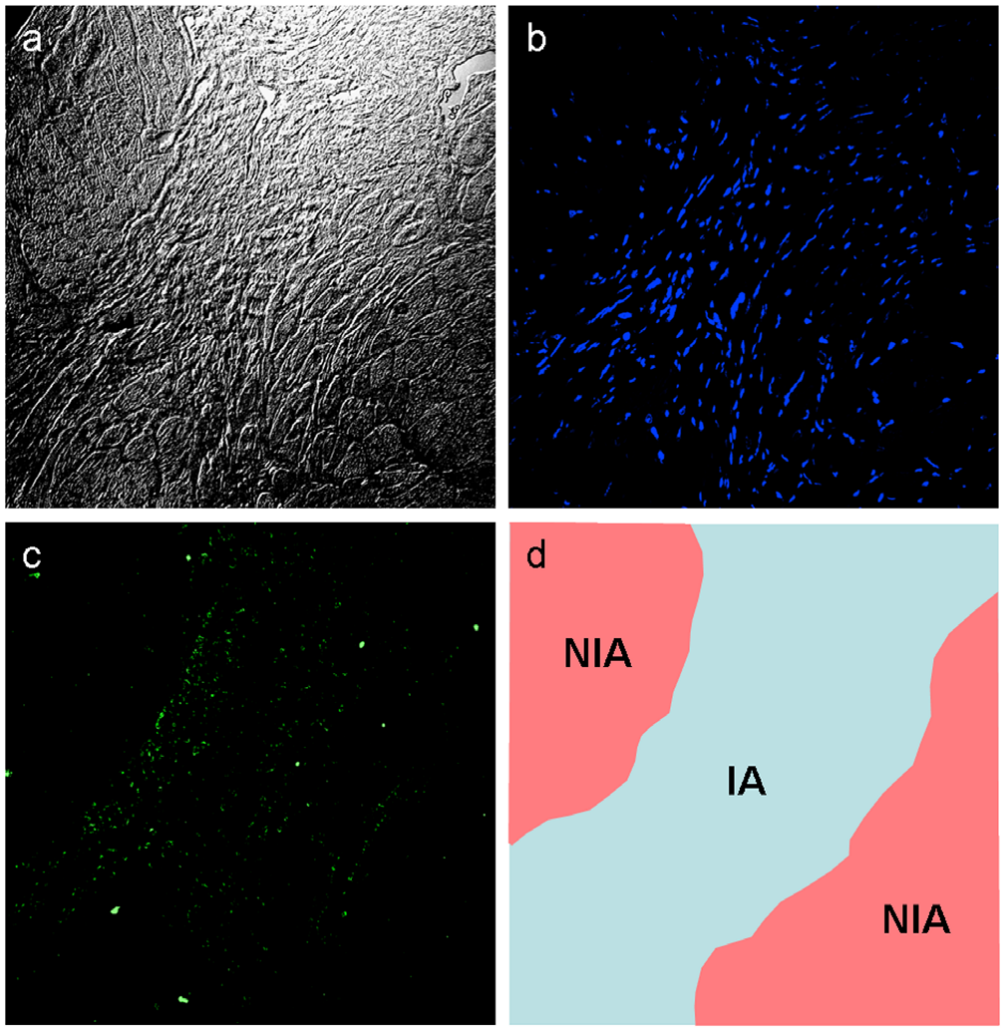
Immunohistochemistry of MMP-9 in infarcted myocardium. (a) A DIC image of the left ventricular infarct area. (b and c) The same fields stained with Hoechst 33258 to indicate the position of nuclei (b) and with a rabbit anti mouse MMP-9 antibody to indicate the localization of MMP-9 (c). (d) A schematic drawing of the field observed. IA and NIA represent infarct and non-infarct area, respectively.

**Figure 7:**
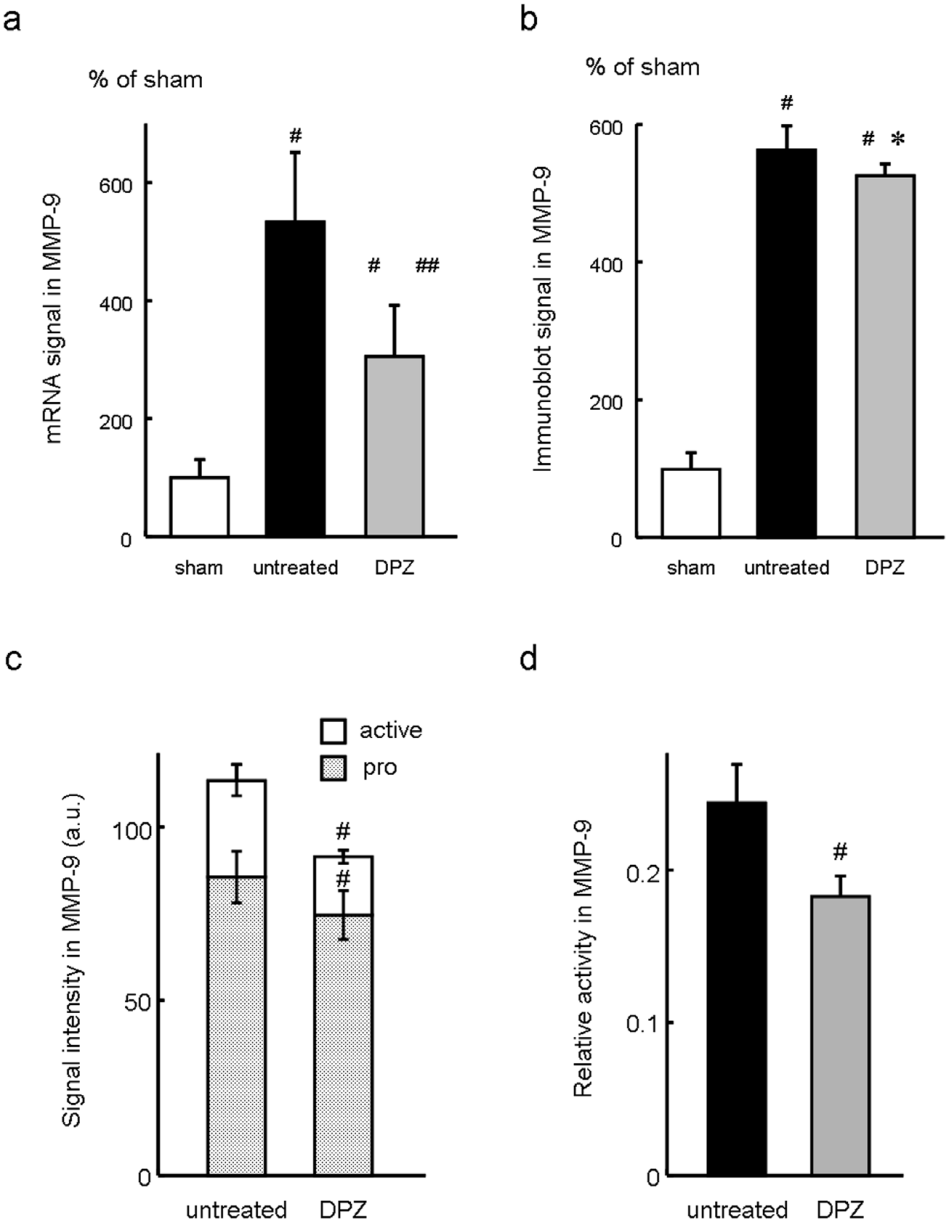
MMP-9 expression and its enzymatic activity in infarcted myocardium. MMP-9 expression in the left ventricular infarct area was compared among sham, untreated and donepezil-treated groups in mRNA (a) and protein level (b) at 3 days after myocardial infarction. (a) Compared to sham group (sham), MMP-9 mRNA in infarcted groups was significantly increased (untreated and DPZ). The increase of MMP-9 mRNA was significantly attenuated in the donepezil-treated group compared to the untreated group. #, *P<0.01* versus sham group. ##, *P<0.01* versus untreated group. (b) Compared to sham group (sham), MMP-9 protein in infarcted groups was significantly increased (untreated and DPZ). The increase of MMP-9 was slightly but significantly attenuated in the donepezil-treated group compared to the untreated group. #, *P<0.01* versus sham group. *, *P<0.05* versus untreated group. Enzymatic activity of MMP-9 in the left ventricular infarct area was evaluated by gelatin zymography (c and d) at 3 days after myocardial infarction. (c) Compared to the untreated group (untreated), signal intensity in both pro and active forms of MMP-9 were significantly decreased in the donepezil-treated group (DPZ). #, *P<0.01*. (d) Relative activity (active/total MMP-9) was calculated and was significantly decreased in the donepezil-treated group (DPZ) compared to the untreated group (untreated). #, *P<0.01*.

As shown in Figure 6, immunofluorescence microscopy revealed that MMP-9 signals were localized at a region of an infarct left ventricle or a border zone between infarcted and non-infarcted myocardium where a large number of nuclei were observed, suggesting that MMP-9 was produced by inflammatory cells including macrophages which infiltrated into infarct area after MI. Therefore, the effect of donepezil on the MMP-9 expression in tissues of left ventricular infarct area was examined at 3 days after MI. RT-PCR analysis showed that MMP-9 mRNA level was significantly increased (untreated, 532.60±119.24%, n = 11, *P<0.01*) compared with sham group (100.00±30.00%, n = 6), and that the increase of MMP-9 mRNA level was significantly attenuated in the donepezil-treated group (DPZ, 306.00±86.52%, n = 11, *P<0.01*) compared with the untreated group (Figure 7a). At the same time point, Western blot analysis showed that MMP-9 protein level was significantly increased in infarcted groups (untreated, 562.68±23.11%, n = 5, *P<0.01* and DPZ, 525.75±16.31%, n = 5, *P<0.01*) compared with sham group (100.00±23.11%, n = 3), and was slightly but significantly decreased in the donepezil-treated group compared with the untreated group (*P<0.05*, Figure 7b). Enzymatic activity of MMP-9 in the left ventricular infarct area was evaluated by gelatin zymography at 3 days after MI (Figures 7c and d). Compared to the untreated group (untreated), signal intensity in both pro- and active-forms of MMP-9 in the donepezil-treated group (DPZ) were significantly reduced (pro MMP-9; untreated, 85.4±7.3 in a.u., n = 7 and DPZ, 74.5±6.9 in a.u., n = 7, *P<0.01*, active MMP-9; untreated, 27.7±4.5 in a.u., n = 7 and DPZ, 16.7±1.9 in a.u., n = 7, *P<0.01*, Figure 7c). Furthermore, relative enzymatic activity of MMP-9 was expressed as the ratio of active-form to total (pro- + active-form) MMP-9 signal intensity, and was significantly decreased in the donepezil-treated group compared to the untreated group (untreated, 0.24±0.02, n = 7 and DPZ, 0.18±0.01, n = 7, *P<0.01*, Figure 7d).

## Discussion

Since we reported that the chronic vagal stimulation markedly improved the cardiac function and decreased mortality in a post-ischemic rat model of heart failure [3], we have focusing on elucidating the cellular and molecular mechanisms behind the ACh-induced cardioprotection. As results, vagal stimulation and ACh have been shown to 1) prevent the loss of functional gap-junction channels and protect hearts against ischemia-induced lethal arrhythmias [4], 2) protect cardiomyocytes against hypoxia through the PI3K/Akt/HIF-1α pathway [5], 3) keep cell-cell communication by preserving the gap-junction protein level during hypoxia through inhibition of the connexin 43 degradation pathway [34], and 4) prevent a reperfusion-induced collapse in mitochondrial transmembrane potential by inhibition of mitochondrial permeability transition pore opening [35]. In the present study, to test whether or not an acetylcholinesterase inhibitor donepezil reproduced the cardioprotective effect of the vagal stimulation or ACh, we investigated the effect of donepezil on inflammatory response and mortality after MI. As a result, it was shown that donepezil inhibits macrophage MMP-9 and reduces the risk of cardiac rupture during the acute phase of MI. These findings, taken together with our earlier studies [13]–[15], indicate that donepezil would be a novel pharmacotherapeutic drug for heart failure patients.

Murine MI is a good model for exploration of the molecular processes of ischemia-induced tissue injury, because the postinfarct inflammatory response shares common characteristics with higher mammalian species [36] and the murine ventricular free wall often ruptures within a week after the onset of MI, like humans [24], [37], [38]. The postinfarct inflammation has been recognized as an important feature of the cardiac remodeling after MI. A number of studies have reported that MMPs play important roles in the left ventricular remodeling, and inhibiting the enzymatic activity of MMPs prevented the progress of cardiac dysfunction and improved survival following MI [26], [29], [39]. Among the members of MMP family, MMP-9 seems to be a key enzyme in early myocardial remodeling and fatal cardiac rupture during an acute phase of MI [25], [27]. After the onset of MI, macrophage infiltrates into infarcted myocardium and expresses MMP-9 to degrade extracellular matrix [40], [41]. The present study demonstrated that, in *in vitro* study, donepezil inhibited MMP-9 in LPS-treated macrophages (Figures 1, 2, 3, 4) and that, in *in vivo* study, donepezil attenuated the inflammatory cell infiltration into the infarcted myocardium and decreased the expression and the enzymatic activity of MMP-9 at the infarct area (Figure 7), thereby reduced the risk of cardiac rupture during the acute phase of MI (Table 3). These results indicate that donepezil is a possible candidate agent for affecting the postinfarct inflammation and the extracellular matrix remodeling. Macrophage MMP-9 in culture medium was not affected by coincubation with donepezil (100 µM) at 37°C for 3 hrs (data not shown), suggesting that donepezil might not possess degradation activity toward MMP-9 but attenuate macrophage MMP-9 protein expression through certain receptors not yet identified. To clarify a donepezil-binding receptor and an intracellular signaling pathway downstream of the receptor, a future study should be conducted.

Histological analysis on infarcted heart sections showed that left ventricular wall thinning and right ventricular hypertrophy were attenuated in the donepezil-treated group (Table 2), however, without significant differences of the heart to body weight ratio and the infarct size, suggesting that donepezil seems to have a mild anti-remodeling effect on the infarcted heart at this time point (3 days postinfarction). Masson's trichrome staining on the hearts 3 days after MI also supported this result, because collagen fibers were not distributed and cardiac myocytes still existed throughout the infarct area (data not shown). These observations indicate that the extracellular matrix turnover, i.e., the synthesis and degradation of collagen, to replace necrotic cardiomyocytes has not yet occurred, and LV remodeling would progress later. Nevertheless, in this study, cardiac rupture occurred frequently within 4 days after the onset of MI, and donepezil treatment reduced the risk of the lethal event. When ruptured, the wall perforation tended to occur at the infarct border zone (Figures 5c and d) where a large number of inflammatory cells invaded (Figure 6b). The number of inflammatory cells at the area was significantly lower in the donepezil-treated group than the untreated group (Table 2), indicating that donepezil attenuated the infiltration of the inflammatory cells into the infarct border zone after MI. Although it is still unclear whether or not donepezil inhibited MMP-9 production of the infiltrated inflammatory cells at the infarct border zone, it is conceivable that donepezil exerted the anti-rupture effect by inhibiting the inflammatory cell infiltration at the infarct border zone and possibly by retaining the local strength of the infarct ventricular wall.

In this study, the expression of MMP-9 mRNA in the left ventricular infarct area at 3 days after MI was reduced by donepezil in approximately half compared with that in the untreated group, whereas this was not accurately reflected in the protein level at this time point (Figures 7a and b). This might be due to the time difference in the process of transcription to translation, i.e., the inhibitory effect of donepezil on the de novo synthesis of MMP-9. In any case, the donepezil-treated mice showed a significantly lower incidence of cardiac rupture than the untreated mice (Table 3), accompanied by a reduced expression and enzymatic activity of MMP-9 in the left ventricular infarct area (Figure 7). Further studies are required to elucidate whether or not donepezil has effect on other types of MMP family members such as MMP-2 and MMP-13, and on their endogenous inhibitors, tissue inhibitor of matrix metalloproteinases (TIMPs). The effects of donepezil on the other cells including neutrophils, another source of MMP-9 [42], and cardiomyocytes should also be investigated.

Chronic effect of donepezil on ischemic heart failure has been also investigated in rats, demonstrating that donepezil improved the long-term survival through the prevention of postinfarct ventricular dysfunction and cardiac remodeling [13], [43], [44]. Although cardiac rupture has not so far been reported in rat models with MI as well as other laboratory animals, such as rabbits, dogs, pigs and sheep [45], pharmacological intervention of the MMPs attenuates the postinfarct remodeling [26], [28], [46], [47]. Furthermore, donepezil pretreatment (100 µM) inhibited the LPS-induced increase of MMP-9 secretion even in rats macrophage (data not shown). Therefore, the inhibitory effect of donepezil on macrophage MMP-9 is also involved in protecting from rat cardiac remodeling during the acute phase of MI. Studies to determine the long-term effects of donepezil on inflammatory responses after MI are also needed.

It is well known that a large dose of donepezil has a heart-rate slowing effect [48]. The dose of donepezil administrated to mice in the present study was approximately 50 times larger than the therapeutic doses used for Alzheimer's disease patients in clinical setting. However, the selected doses of donepezil lowered an incidence of cardiac rupture during acute phase of MI without slowing heart rate, indicating that the anti-rupture effect of donepezil was not brought by its bradycardiac action. Although the daily dose at 5.0 mg/kg has been extensively used in animal study with donepezil, it should be considered whether or not the clinical dose for donepezil also has a beneficial effect on heart failure comparably with the higher dose. It was shown that the oral administration of donepezil at dose of 0.5 mg/kg/day prevented the progression of left ventricular dysfunction on rat MI heart (unpublished data), suggesting that donepezil at dose of 5.0 mg/kg/day is not necessarily required for the treatment of heart failure. Actually, our recent retrospective cohort study showed that donepezil-treated patients with Alzheimer's disease had reduced cardiovascular mortality [49].

It has been reported that the cholinergic anti-inflammatory pathway can directly modulate the systemic response through ACh by attenuating the release of inflammatory cytokines from macrophages. In the present study, donepezil inhibited MMP-9 expression in LPS-treated macrophages, but not tumor necrosis factor (TNF). The inhibitory effect of donepezil on macrophage MMP-9 could not be reproduced by ACh (Figure 2a) or other kinds of acetylcholinesterase inhibitors such as physostigmine and galanthamine (data not shown). Experimentally, ACh was shown to inhibit TNF release from LPS-stimulated macrophages [1], however, in our study, ACh failed to inhibit TNF release (data not shown). This discrepancy between our results and those of Borovikova et al. [1] might be derived from different cell sources and culture conditions. Moreover, neither a muscarinic ACh receptor blocker, atropine, nor a nicotinic ACh receptor blocker, mecamylamine, attenuated the inhibitory effect of donepezil on the MMP-9 release (Figure 2b). These results indicate that the specific characteristics of donepezil would be independent of its acetylcholinesterase inhibition. Recently accumulating evidence strongly suggests that donepezil possesses a direct cytoprotective activity, independent of its acetylcholinesterase-inhibitory mechanism [12], [50]–[52]. Our previous study using an ischemic hindlimb model of α7 nicotinic receptor-deleted mice demonstrated the beneficial effect of donepezil on acceleration of angiogenesis, suggesting that donepezil rather exerts its specific effect independent of α7 nicotinic receptors [15]. Further studies focusing on the pharmacological properties of donepezil which directly affect cells via mechanism other than acetylcholinesterase inhibitory action are needed.

In summary, the results of the study reported here suggest that donepezil inhibits MMP-9 in macrophages which infiltrate into the infarcted myocardium, thereby contributes at least in part to the reduction of the risk in left ventricular free wall rupture during the acute phase of MI. Although donepezil is clinically used as a central type inhibitor of neural acetylcholinesterase, the donepezil-induced cardioprotection observed in this study may not be due to its pharmacological property of acetylcholinesterase inhibition. Understanding of the cellular and molecular mechanism of the donepezil-induced cardioprotection would help to establish a novel therapeutic strategy in the prevention and treatment against heart failure.
